# Neurotrophic keratopathy in childhood: advances in understanding of pathogenesis and management

**DOI:** 10.1038/s41433-026-04278-7

**Published:** 2026-02-05

**Authors:** Jana Jiang, Christopher B. J. Ashton, Daniel F. P. Larkin

**Affiliations:** 1 External Diseases Service, Moorfields Eye Hospital, London, UK; 2 Eye Center, Medical Center, Faculty of Medicine, University of Freiburg, Freiburg, Germany; 3 NIHR Moorfields Biomedical Research Centre, Moorfields Eye Hospital, London, UK

**Keywords:** Paediatrics, Corneal diseases, Hereditary eye disease, Eye manifestations

## Abstract

Neurotrophic keratopathy (NK) is an uncommon corneal disorder caused by trigeminal nerve dysfunction, leading to loss of ocular surface sensation, impaired corneal epithelial maintenance, and possible progressive stromal lysis. NK is of added potential visual significance in children on account of the risk of amblyopia resulting from stromal opacification. Unlike acquired NK in adult-onset disease, NK in childhood is frequently congenital or inherited, linked to genetic pain insensitivity syndromes, cranial dysinnervation disorders or broader developmental anomalies. Visual function can be well preserved in affected eyes in many children with supportive management or specific medical and surgical interventions directed at modulating sensory nerve function. A modification of the existing classification of NK stages is proposed to incorporate those eyes in which there is no detectable corneal sensation but otherwise normal eye examination, a phenotype not infrequently encountered in early NK in childhood.

## Introduction

Neurotrophic keratopathy (NK), also known as neurotrophic keratitis, is an uncommon corneal disorder caused by the malfunction of the sensory trigeminal nerve. This results in disruption of sensory innervation of the ocular surface but also compromise of the integrity of the corneal epithelium and maintenance of its protective functions. NK can progress from subclinical epithelial abnormalities to persistent ulceration, stromal lysis, secondary corneal infection and perforation.

A distinguishing characteristic of NK in childhood compared to NK acquired in later life is that the aetiology is predominantly inherited or congenital. As in adults, NK, in which onset is in childhood, is sight-threatening. However there are clinical characteristics which significantly differ, including the additional risk of amblyopia caused by corneal stromal opacification, diagnostic challenges in non-verbal children, and specific management approaches such as neurotisation for bilateral cases. Since the initial description of corneal nerves by Schlemm [1], the discovery of nerve growth factor (NGF) by Levi-Montalcini [2] and the description of eye manifestations of familial dysautonomia by Riley and coauthors [3], recent advances in the investigation of corneal innervation and therapeutics in eyes with NK have required new classifications of the causes of NK.

## Anatomy and neurochemistry

The cornea contains the highest density of sensory nerves in man, which play a critical role in sensory perception, corneal epithelial homeostasis and ocular defence, all of these contributing to transparency and normal visual acuity. Sensory innervation primarily originates from the ophthalmic branch of the trigeminal nerve [4]. Most of the nerve fibres are nociceptive myelinated Aδ and unmyelinated C fibres, specialised for detecting mechanical, thermal and chemical stimuli [5].

### Central and brainstem anatomy of the trigeminal nerve

The trigeminal nerve’s central anatomy consists of a complex arrangement of ganglia, nerve roots, nuclei, and associated fibre pathways. A key structure is the trigeminal (Gasserian) ganglion, located within a pouch formed by the dura mater on the petrous apex of the temporal bone. This ganglion houses the cell bodies of all trigeminal sensory neurones [6, 7]. The central processes of these pseudo-unipolar neurones exit the ganglion from its dorsal side, merging into the sensory nerve root which enters the brainstem laterally at a mid-pontine level [8]. Within the brainstem, afferent sensory fibres from the trigeminal nerve connect with three sensory nuclei: the mesencephalic nucleus, the principal sensory nucleus and the spinal trigeminal nucleus. Situated in the mid-pons, the principal sensory nucleus is the largest among these trigeminal nuclei [6]. Its ventrolateral subnucleus receives sensory information from the ophthalmic and other branches of the trigeminal nerve [6, 9].

### Trigeminal sensory fibres to the limbal plexus

Peripheral sensory fibres innervating the cornea originate from pseudo-unipolar neurones in the trigeminal ganglion described above. These fibres travel through the ophthalmic division, specifically via the nasociliary branch, which gives rise to the long ciliary nerves that supply the peripheral cornea [10]. The long ciliary nerves travel in the suprachoroidal space towards the anterior segment, where they branch into bundles that, along with nerve fibres from the subconjunctival plexus, establish the limbal plexus at the corneoscleral limbus [11].

### Stromal nerves, sub-basal nerve plexus and intraepithelial free nerve endings

Radially arranged stromal nerve trunks arise from the limbal plexus and enter at a mid-stromal level peripherally [4]. The nerve trunks lose their perineurium and myelin sheaths shortly after entering the cornea to ensure optical transparency and split into numerous smaller branches as they advance anteriorly [4]. Stromal nerves then penetrate Bowman’s layer to create the sub-basal nerve plexus, distinguished by its whorl-like configuration near the inferonasal corneal apex [12, 13]. Corneal epithelial nerve endings originate from this plexus, extending vertically through the epithelial layers and terminating in the superficial epithelium as free nerve endings [10].

### Trophic function of corneal nerves and nerve growth factor (NGF)

Beyond their anatomy, corneal nerves not only provide sensory input but also exert critical trophic effects that support epithelial integrity and regeneration. Corneal nerves release neurotransmitters including substance P (SP), calcitonin gene-related peptide (CGRP) and NGF, which promote epithelial cell proliferation, differentiation, migration and adhesion [14–17]. NGF, discovered in 1951 [2], consists of three subunits - α, β, and γ - with the β-subunit responsible for nerve growth-stimulating activity [18]. Like other neurotrophic proteins, NGF is synthesised as a proneurotrophin and must undergo proteolytic cleavage by extracellular molecules (e.g. matrix metalloproteinases) to become functionally active [19]. While all neurotrophins bind to the low-affinity receptor p75NTR, NGF specifically acts through the high-affinity receptor TrkA [20, 21]. Upon binding to TrkA, NGF activates intracellular signalling cascades that drive neuronal survival, differentiation, and axonal growth [22]. Beyond neural development, NGF-TrkA binding plays a critical role in corneal wound healing by promoting proliferation and migration of epithelial and stromal cells [23].

## Causes of neurotrophic keratopathy in childhood

With a spectrum of conditions causing partial or complete corneal anaesthesia [24, 25], the aetiology of neurotrophic keratopathy differs in children and adults. While in adults NK is acquired and most commonly secondary to herpetic infections, diabetes mellitus and surgery or neurological disorders, in children NK is usually congenital or inherited (Table 1) [26].

**Table 1:** Congenital and inherited causes of neurotrophic keratopathy.

Congenital	Inherited
**Non-syndromic** • Congenital trigeminal anaesthesia	**Genetic pain loss disorders / Hereditary sensory and autonomic neuropathies (HSAN)**^**a**^
**Axial mesodermal dysplasia complex (AMDC)** • Oculo-Auriculo-Vertebral Spectrum (Goldenhar Syndrome)	• Type III (Familial dysautonomia) • Type IV • Type V
**Congenital Cranial Dysinnervation Disorders (CCDD)** • Pontine tegmental cap dysplasia • Möbius Syndrome	

^a^All types of hereditary sensory and autonomic neuropathies (HSANs) can be associated with congenital corneal anaesthesia. Those listed are HSAN III, the best characterised type, as well as HSAN IV and V, which are associated with impaired neurotrophin signalling [35].

### Inherited corneal anaesthesia

Inherited trigeminal anaesthesia is a component of the genetic pain loss disorders, an umbrella term encompassing both congenital insensitivity to pain (CIP) and hereditary sensory and autonomic neuropathy (HSAN). These have recently been reclassified and divided based on age of onset, with congenital cases often classified as CIP and cases presenting later as HSAN [27]. However, this distinction remains fluid, as overlapping clinical features and evolving terminology complicate strict categorisation. These conditions can stem from autosomal recessive mutations that generally present in infancy or from autosomal dominant variants that may appear later and are characterised in some by incomplete penetrance [27, 28].

The hallmark of these rare disorders is significantly reduced pain perception due to aberrant small nerve fibre development [28]. They present with variable severity and systemic symptoms, such as dysautonomia and self-mutilating behaviour. Corneal sensation is typically absent, leading to corneal epithelial defects and infection in early-onset forms [27], which can progress to the later stages of neurotrophic keratopathy if left untreated. The following section focuses on the HSAN subtypes most commonly associated with corneal anaesthesia.

#### Hereditary sensory and autonomic neuropathy type III (HSAN III)

HSAN III, also known as familial dysautonomia or Riley-Day syndrome [3], is an autosomal recessive disorder primarily affecting individuals of Ashkenazi Jewish descent, with an incidence of approximately 1 in 3600 live births [29]. It is characterised by a distinctive combination of sensory deficits, including corneal anaesthesia, and autonomic abnormalities. While patients do exhibit reduced pain and temperature perception, these sensory deficits are typically milder than in other HSAN types and rarely result in self-mutilation [28]. A key feature, termed dysautonomic crisis, involves severe nausea, vomiting, sweating and sudden cardiovascular changes, which can be cyclical or triggered by physical or emotional stress [28].

HSAN III primarily arises from a single point mutation in the elongator complex protein 1 (*ELP1*, formerly *IKBKAP*) gene, with over 99% of affected individuals homozygous for this variant [30]. ELP1 protein functions as a scaffolding protein within the elongator complex; its reduction in neurones causes disrupted neuronal development and progressive degeneration of both sensory and autonomic neurones [31, 32]. Beyond the absence of tears and corneal anaesthesia, ocular findings in HSAN III include optic neuropathy in the second and third decades, and abnormal ocular motility [33]. On account of the very high degree of homozygosity of the founder *ELP1* mutation, a gene replacement therapy has been developed and licensed for intravitreal injection to prevent retinal ganglion cell loss and optic neuropathy [34].

#### Hereditary sensory and autonomic neuropathy type IV (HSAN IV)

HSAN IV, or congenital insensitivity to pain with anhidrosis (CIPA), is the second most common type of HSAN. Its hallmark is anhidrosis, which may range from reduced to completely absent sweating, often leading to febrile seizures [28, 35]. In contrast to HSAN III, other autonomic disturbances are less common [28]. Most patients exhibit intellectual disabilities and developmental delays [28, 36].

Inheritance is autosomal recessive, with around 50% of the cases linked to consanguinity [28]. HSAN IV is caused by mutations in the *NTRK1* gene, which compromises the production of a functional TrkA receptor, essential for NGF-mediated signalling and the survival of sensory and autonomic neurones [28, 36]. This is directly relevant to treatment options in these cases, as exogenous therapeutic NGF (cenegermin), discussed below, requires functional TrkA receptors. In patients with HSAN IV, punctate corneal epitheliopathy is typical [37], and preservation of the blink reflex is strongly associated with better visual acuity [38].

#### Hereditary sensory and autonomic neuropathy type V (HSAN V)

HSAN V is a rare autosomal recessive disorder caused by mutations in the *NGF* gene, which encodes nerve growth factor beta (NGFβ). In the neurotrophin signalling pathway, NGF binds to the high-affinity receptor TrkA (encoded by NTRK1), a process essential for sensory neuron survival and target innervation [27, 39]. HSAN V presents as congenital insensitivity to pain with relatively preserved autonomic function and less pronounced anhidrosis compared to HSAN IV [40, 41].

HSAN IV and HSAN V both highlight the critical importance of the neurotrophin signalling pathway in neuronal development [27]. In HSAN IV, the disruption occurs at the receptor level (TrkA), whereas in HSAN V it arises from mutations in the ligand (NGF).

### Congenital, non-inherited, corneal anaesthesia

#### Non-syndromic

Isolated congenital corneal anaesthesia, without systemic associations, is rare and in most cases bilateral. Presentation is typically at age 2–5 years with painless infectious keratitis in one eye [25]. Most commonly, corneal anaesthesia is caused by underlying congenital trigeminal anaesthesia, manifest as anaesthesia in a variable area in the distribution of the ophthalmic division of the trigeminal nerve [24, 42]. Trigeminal anaesthesia in turn may occur as an isolated phenomenon or as a consequence of trigeminal nerve aplasia or hypoplasia [24, 42].

### Congenital cranial dysinnervation disorders (CCDDs)

Congenital, non-progressive neuromuscular conditions characterised by abnormal innervation and development of the ocular and facial muscles are collectively referred to as congenital cranial dysinnervation disorders (CCDDs) [43]. One or more cranial nerves, both motor and sensory, may be underdeveloped or absent, often accompanied by secondary muscle pathology and additional orbital or bony structural anomalies [43]. Reduced corneal sensitivity has been reported in two of these disorders: Pontine Tegmental Cap Dysplasia (PTCD) and Möbius Syndrome (MbS).

#### Pontine tegmental cap dysplasia

**Fig. 1:**
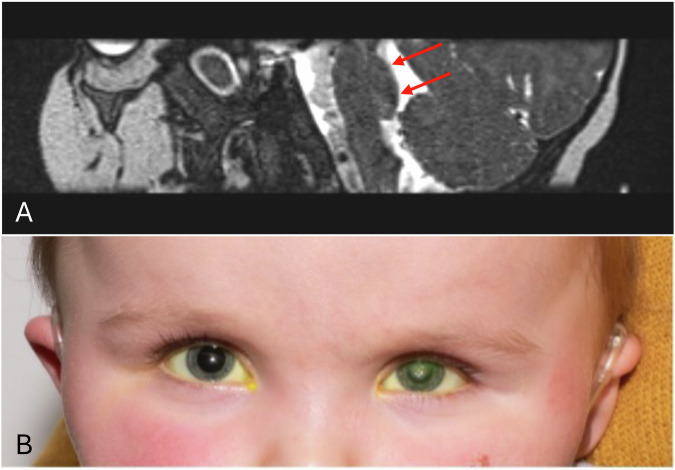
Pontine tegmental cap dysplasia. **A** Pons sagittal T2 weighted magnetic resonance image: ventral hypoplasia and appearance of a rounded “cap” (red arrows) projecting into the 4th ventricle. **B** Two-year old with bilateral corneal anaesthesia and left corneal opacity. Magnetic resonance imaging demonstrated pontine tegmental cap dysplasia but also associated absence of auditory and vestibular nerves.

Originally reported in 2007 by Barth et al, the defining radiological finding in PTCD is the “tegmental cap”, which describes the vaulted pontine tegmentum projecting into the fourth ventricle (Fig. 1A) [44]. Additional brainstem and cerebellar malformations have been reported in some patients [44]. While cochlear nerves are affected in all reported patients, involvement of other cranial nerves varies between patients [44, 45]. NK is the commonest eye feature, and optimum management to minimise corneal opacity is especially important in these patients with deafness (Fig. 1B) [46]. The pathogenesis of PTCD remains unclear [44, 47]. So far, genetic testing has not identified a definitive causative gene or mutation for this condition, which occurs sporadically in both sexes and shows no clear familial pattern [44, 45, 47].

#### Möbius Syndrome

Möbius syndrome is characterised by unilateral or bilateral facial weakness and impaired ocular abduction, attributed to underdevelopment of the sixth and seventh cranial nerves [48, 49]. Other cranial nerves may also be affected. Because of lagophthalmos, the cornea is vulnerable to exposure keratopathy and, in a small number of cases, corneal anaesthesia results from involvement of the trigeminal ophthalmic division, further increasing the risk of ulceration and infection [25, 48–50]. Although most cases occur sporadically, familial inheritance has been documented in rare instances [51, 52].

### Axial mesodermal dysplasia complex (AMDC)

Axial mesodermal dysplasia complex (AMDC) encompasses a broader spectrum of congenital malformations than CCDDs, affecting multiple body systems [53]. Depending on the specific syndrome, malformations can involve any major organ system (pulmonary, cardiovascular, gastrointestinal, renal, urogenital, skeletal), often with overlapping features [53–55]. Like CCDD, most AMDC cases are sporadic, and their aetiology is most likely multifactorial, involving genetic and environmental factors [55].

#### Oculo-auriculo-vertebral spectrum (OAVS)

The most frequent AMDC, also known as Goldenhar syndrome, is OAVS and a well-recognised condition in ophthalmology. It is characterised by features such as epibulbar or limbal dermoids, microphthalmia, and colobomas affecting the eyelid, iris, or choroid. Corneal anaesthesia is found in some cases, in which underlying trigeminal nerve aplasia has been identified [56]. Ear abnormalities are prominent in OAVS, including preauricular tags, microtia, and external auditory canal anomalies. Systemic involvement is also common, with congenital heart defects being the most frequent, along with renal, skeletal, and central nervous system malformations [57].

## Assessment of suspected neurotrophic keratopathy in children

Diagnosis and investigation of NK in children requires a systematic approach due to the challenges of examination in very young children and the potentially limited ability of young patients to report symptoms. Assessment includes the following.

### History

The case history may identify potential aetiological factors specific to childhood onset. General health history is more important than in adult NK, particularly in congenital NK, which may be associated with developmental delay and intellectual disability, and the family history may indicate neurological or eye disorders that affect corneal sensation.

### Eye examination

NK diagnosis is based on the reduction or absence of corneal sensation. An estimation can be made using a cotton wool pledget, easily performed in children but not standardised or quantifiable. The Cochet-Bonnet aesthesiometer remains the gold standard for measuring corneal sensitivity [58]. Its nylon filament touches the cornea to elicit a response. The filament is shortened from 60 mm to 10 mm, giving an increase in pressure (from 0.4 to 16 g/mm^2^) until the patient confirms a response. The filament is advanced until a five-degree bend is observed [59]. If reduced corneal sensation is confirmed, sensation in the conjunctiva and periocular skin on both sides should be tested and is frequently found to be reduced. Abnormalities of lid position, voluntary lid closure and any strabismus may indicate associated cranial nerve deficits. Schirmer testing should always be performed.

Slit-lamp examination identifies any cornea abnormality, including epithelial defects on fluorescein staining, stromal scarring, vascularisation or thinning, used to stage keratopathy. The Mackie classification categorises NK into three stages [60]. Stage 1 is characterised by corneal epithelial hyperplasia and irregularity, punctate epitheliopathy, decreased tear film break-up time, variable intraepithelial opacity and superficial vascularisation. Progression to stage 2 is indicated by persistent epithelial defects, usually oval with rolled and smooth edges, variable stromal oedema and Descemet’s membrane folds. Features in stage 3 are corneal ulceration with stromal lysis that may progress to perforation. This classification was proposed by Mackie without reference to NK in childhood, in which it is not uncommon that there is no detectable corneal sensation but no additional abnormal eye findings: normal epithelium, tear film, transparent cornea and absence of any fluorescein staining. NK in such eyes should be classified as stage 1 in a modified classification, with re-classification of Mackie stages 1, 2 and 3 as stages 2, 3 and 4, respectively (Table 2). With the introduction of novel medical and surgical interventions for NK described below, this modified classification enables more descriptive categorisation of NK phenotypes for comparison of eyes and patient groups in studies and assessment of treatment outcomes.

**Table 2:** Classification of slit-lamp corneal findings in childhood neurotrophic keratopathy (modified from Mackie [60], [80]).

Neurotrophic keratopathy stage	Fluorescein staining	Other features
1	None	Normal slit-lamp examination, other than ocular surface anaesthesia
2	Punctate erosions	Variable superficial opacity
3	Epithelial defect	Rolled epithelial edges Variable superficial opacity Variable superficial vascularisation No stromal loss
4	Epithelial defect	Superficial vascularisation Stromal thinning

In vivo confocal microscopy (IVCM) enables high-quality non-invasive imaging of the cornea and can be performed in children with sufficiently steady fixation. Major reductions in sub-basal nerve plexus (SBNP) nerve fibre density, fibre length and branch density have been reported in congenital and inherited corneal anaesthesia, in addition to significant increases in populations of immune dendritiform cells adjacent to the SBNP [61, 62].

Although the diagnosis of NK is primarily clinical, radiological investigations of the brainstem may be necessary to identify an underlying diagnosis, such as pontine tegmental cap dysplasia.

## Management of suspected neurotrophic keratitis in children

Treatment of NK is based on the clinical findings and is determined according to keratopathy stage (Table 3). The goal of treatment is to protect the ocular surface, and in circumstances of ulceration, to promote ocular surface repair and prevent progression to perforation. The added imperative in younger children is to prevent amblyopia caused by corneal stromal vascularisation, opacification and thinning.

**Table 3:** Stage-based prevention and treatment of corneal ulceration in neurotrophic keratopathy.

Stages 1, 2: Prevention of ulceration
• Lubricants, punctal plugs
• Scleral contact lenses
• Lateral tarsorrhaphy
• Neurotisation procedures^a^
**Stages 3, 4: Treatment of ulceration**
• Botulinum toxin-induced ptosis
• Tarsorrhaphy
• Serum drops
• Human insulin 1U/ml drops
• Recombinant NGF (cenegermin), or mimetics

^a^Although neurotisation is undertaken to prevent recurrences of stage 3 or 4 NK, the stage at surgery is frequently stage 2.

Those children with NK but intact epithelium or stable mild punctate corneal epitheliopathy are usually adequately managed with supportive medical management, including lubricants, frequent monitoring and advice to parents to arrange urgent examination in the event of a red eye, which might be indicative of corneal infection.

### Cornea neurotisation

In this procedure, a healthy donor nerve segment is directly transferred to restore cornea sensation and trophic function. Direct and indirect techniques have been described. The direct technique described by Terzis and coauthors involves harvesting of the contralateral supraorbital and supratrochlear nerve branches and tunnelling them across the bridge of the nose to insert around the limbus of the anaesthetic cornea [63]. This technique denervates the contralateral forehead and scalp, requires a large incision resulting in donor site morbidity, facial scarring and is not applicable in bilateral disease. A less invasive technique using endoscopic harvesting of the supraorbital nerve was described in 2014, in which 10–15 cm of the sural nerve is harvested and coapted end-to-side with the supraorbital and/or supratrochlear nerves through small forehead incisions, following which the graft nerve fascicles are tunnelled into the cornea [64]. This indirect neurotisation method is especially appropriate in children with bilateral NK. Direct neurotisation results in quicker restoration of cornea sensation compared with indirect neurotisation, following which sensation becomes evident within six months and continues for up to two years [65]. IVCM post-neurotisation has shown axonal sprouting at 3 months and definitive nerves at 8 months [66].

The place of neurotisation and the timing of surgery is, in most cases, in the setting of recurrent epithelial breakdown, in which the procedure is performed to prevent further ulceration and stromal opacification. A meta-analysis of corneal neurotisation of all age groups showed that the procedure provides significant clinical improvement in visual acuity, NK staging, and corneal sensation. Indirect neurotisation was associated with greater cornea sensation compared to direct neurotisation. In the meta-analysis, all congenital NK cases treated by neurotisation have been treated by the indirect method [67]. Congenital NK patients had lower levels of visual acuity post-operatively compared to cases of acquired NK, but this could be partially due to shorter follow-up periods and difficulty in visual acuity testing in children [67]. The consideration specific to paediatric NK is the question of timing in relation to visual development. Further studies of corneal neurotisation methods in children with long-term follow-up will be necessary to determine not only the optimum surgical technique but also the timing of surgery.

### Nerve growth factor and topical serum

A functional role of exogenous NGF has been demonstrated in maintaining cornea homeostasis in vitro, in animal models [68] and in randomised trials in patients. The first approved NGF treatment developed is cenegermin (Oxervate; Dompé Farmaceutici SpA, Milan, Italy), a recombinant human NGF (rhNGF). Efficacy in repair of neurotrophic corneal ulcers in adults was demonstrated in the REPARO and NGF0214 randomised controlled trials over an 8-week treatment period [69, 70]. Cenegermin 0.002% is approved for use in NK in patients over the age of 2 years.

There are several case reports that demonstrate cenegermin to be safe [71–77]. In the largest paediatric case series, Hatcher et al. reported eight patients, all of whom had previously been unsuccessfully treated with other therapies [71]. In four of the eight eyes, NK was congenital and, in the others, acquired. Five had improved corneal healing, and two had improved best corrected visual acuity. Two patients had no clinical improvement despite therapy, and one with progressive corneal thinning leading to emergency penetrating keratoplasty. It is difficult to draw conclusions from such retrospective reports of uncontrolled cases in a small number of patients. There are two further caveats. First, on a pharmacological basis, cenegermin would not be expected to be effective in all those with congenital NK because NGF activates tyrosine kinase TrkA and p75 receptors. HSAN type IV is caused by mutations in the neurotrophic tyrosine receptor kinase (*NTRK1*) gene, which encodes the TrkA receptor [28]. Therefore, the effectiveness of cenegermin would be limited in these patients due to the absence of TrkA receptors. Second, autologous serum has been effective in treating NK and has been reported to contain substance P, insulin-like growth factor and NGF [78]. Autologous serum is widely available, as are insulin eye drops, which contain insulin-like growth factor and have been used to successfully treat refractory persistent epithelial defects in case series that include patients with NK [79, 80].

## Discussion

Paediatric NK is rare but vision-threatening due to microbial keratitis and the added risk of amblyopia caused by corneal stromal opacification and thinning. Unlike adult-onset NK, which is acquired, childhood cases are mostly congenital or inherited, reflecting diverse genetic and syndromic causes that affect trigeminal innervation and corneal trophic support. Early recognition is therefore essential, if possible, to prevent corneal ulceration, secondary microbial infection and scarring. However, diagnosis is challenging in young children who may not reliably report symptoms or cooperate with examination.

The Mackie classification provides a useful framework for staging of NK but does not enable staging of many of those children with corneal anaesthesia but no other clinical signs. Children may present with absent corneal sensation yet have a clear cornea, normal tear film and no fluorescein staining. This phenotype is not represented in the current NK staging, which we propose as a stage 1, in a modification of the Mackie classification to allow inclusion and classification of such patients with subclinical keratopathy. With new medical and surgical interventions for NK, it will be helpful to incorporate all phenotypes and stages of NK severity in recruitment in trials.

Cellular changes identified by in vivo confocal microscopy provide further insight into the benign clinical course in many childhood cases, in which good vision can be preserved into adult life [26]. In congenital and inherited NK, marked loss of the sub-basal nerve plexus is accompanied by increased dendritic cell density without clinically evident corneal surface inflammation [61]. Similar findings are seen in some eyes with acquired NK [81]. The increased presence of dendritic cells may represent a compensatory biological mechanism supporting corneal integrity and transparency despite sensory loss, by facilitating faster response of enhanced innate immune cell populations in the event of epithelial breakdown and prevention of infection [61].

Management of NK is guided by disease stage and cause. Mild disease can be managed with lubrication and observation, while more severe cases may require trophic support with cenegermin, serum or insulin drops. Corneal neurotisation has emerged as an effective surgical option in children, with indirect techniques particularly suited to bilateral disease. This procedure is the only potentially curative option, an important advantage over non-surgical approaches in young patients with lifelong disease. Significant recent advances for the care of children with NK include improved brainstem MRI for evaluation of trigeminal pathways, and, as therapeutic options, the refinement of neurotisation techniques and the development of NGF as a therapy.

### Future directions in paediatric NK research

One limitation of current research is the scarcity of robust controlled trials on cenegermin or repeated courses of treatment. NGF mimetics now in development are likely to become further therapeutic options for comparative evaluation in controlled trials with cenergemin and/or serum drops. More generally, there is a need for long-term outcome studies on eyes, whether treated by NGF-based approaches or corneal neurotisation, including visual acuity and the influences of the timing of interventions. Longer range research is likely to examine the potential for gene-specific therapies, exemplified by the development of a gene replacement therapy in HSAN type III, and conceivably the investigation of the therapeutic potential of expanded populations of antigen-presenting cells, as suggested by this finding on microscopy in the superficial stroma of eyes of young patients with NK and apparently healthy, secure epithelium.

## Summary

### What is known about this topic

- NK in children is usually caused by trigeminal nerve dysfunction, leading to loss of ocular surface sensation, impaired corneal epithelial maintenance, and possible progressive stromal lysis. Amblyopia is an additional risk in eyes with corneal opacity. NK in children is usually congenital or inherited.

### What this study adds

- This review highlights new information on inherited disorders leading to NK and new classification on underlying causative disorders.
- A revision of the Mackie classification of examination findings in NK is proposed to incorporate findings in childhood with corneal anaesthesia.
